# Retraction: Sulfate activation of wheat straw ash to enhance the properties of high-performance concrete with recycled aggregates and waste tire steel fibers

**DOI:** 10.1371/journal.pone.0346405

**Published:** 2026-04-03

**Authors:** The PLOS One Editors

The *PLOS One* Editors retract this article [1,2] due to concerns about reliance on retracted work cited in References 13, 14, 21, 25, 38, 55, 67, and 75.

All authors either did not respond directly or could not be reached.
